# Rail Flaw Detection via Kolmogorov Entropy of Chaotic Oscillator Based on Ultrasonic Guided Waves

**DOI:** 10.3390/s24092730

**Published:** 2024-04-25

**Authors:** Ziyan Zeng, Jing Wu, Mingfang Zheng, Hongwei Ma

**Affiliations:** 1 School of Mechanics and Construction Engineering, Jinan University, Guangzhou 510632, China; 2 School of Mechanical Engineering, Dongguan University of Technology, Dongguan 523808, China; 3 School of Environment and Civil Engineering, Dongguan University of Technology, Dongguan 523808, China; 4 Guangdong Provincial Key Laboratory of Intelligent Disaster Prevention and Emergency Technologies for Urban Lifeline Engineering, Dongguan 523808, China

**Keywords:** ultrasonic guided waves, chaotic oscillator, Kolmogorov entropy, rail, electromagnetic acoustic transducer

## Abstract

Ultrasonic guided wave (UGW) inspection is an emerging non-destructive testing(NDT) technique for rail flaw detection, where weak UGW signals under strong noise backgrounds are difficult to detect. In this study, a UGW signal identification model based on a chaotic oscillator is established. The approach integrates the UGW response into the critical state of the Duffing system to serve as a disturbance control variable. By evaluating the system’s motion state before and after introducing the UGW response, identification of UGW signals can be realized. Thus, the parameters defining the critical state of Duffing oscillators are determined by $K_{e}$. Moreover, an electromagnetic transducer was specifically devised to enable unidirectional excitation for UGWs targeted at both the rail base and rail head. Experimental studies showed that the proposed methodology effectively detected and located a 0.46 mm notch at the rail base and a 1.78 mm notch at the rail head. Furthermore, $K_{e}$ was directly proportional to the notch size, which could be used as a quantitative index to characterize the rail flaw.

## 1. Introduction

Rail flaw detection is crucial due to the increased likelihood of rail damage from external loads as the service life extends. An accident can lead to significant loss of life and property. Currently, ultrasonic detection is the most widely employed technology for rail flaw detection. The fundamental operation of ultrasonic detection involves using a transducer to excite ultrasonic pulses that characterize and locate internal rail flaws through measured quantities such as the amplitude of the echo signal and time. However, a limitation of ultrasonic testing is that it requires point-to-point scanning of the rail, leading to low detection efficiency and a blind spot at the rail base.

UGW inspection is an emerging NDT technology [1]. Due to the dispersive, multimodal, and attenuating characteristics of UGWs in long range detection, actual sampled guided wave signals often appear as weak signals against a background of strong noise. Scholars have extensively researched signal processing methods for UGWs, including time–frequency analysis [2], such as short-time Fourier transform [3], 2D Fourier transform [4], wavelet transform [5,6], Hilbert–Huang transform [7], empirical modal decomposition [8], Wigner–Ville distribution [9], and artificial neural networks [10], as well as dispersion compensation methods [11], time inversion focusing methods [12], etc. Most of the above methods use noise suppression techniques to reduce the noise of the target signal and the noise signal superimposed on the overlapped signal, which can reduce the sensitivity of damage detection. Furthermore, some state-of-the-art fault detection methods [13,14,15] have been proposed using training data collected with ambient noise in industrial processes.

With the development of nonlinear science, some scholars have begun to study weak signal detection methods based on nonlinear systems, and one of the most representative methods is chaos detection based on chaos theory. Chaos theory discusses the unity of complexity, randomness, and certainty that prevails in nature. Lorenz identified the following three characteristics of chaos [16]: (1) an appearance of randomness, with the actual behavior determined by precise laws; (2) a sensitive dependence on initial conditions; and (3) a sensitive dependence on the intrinsic variability in initial conditions.

Typical models of chaotic dynamics include the Duffing equation [17], the Van-der-pol system [18], logistic mapping [19], and the Loren attractor [20], among which the Duffing equation is a typical model of chaotic dynamics that has garnered significant attention in the field of signal detection, due to its inclusion of a periodic excitation term. Jalilvand [21] examined the impact of frequency, phase, and noise on a weak signal in a Duffing oscillator. Nohara [22] researched the response of the Duffing system when subjected to square wave excitation, assessing its potential for square wave detection. Srinivasan [23] delved into the dynamics of the Duffing equation under sawtooth wave excitation. As research progressed, scholars started incorporating chaotic determination indices into UGW signal detection, Cheng [24] utilized the Poincaré map as a chaos indicator to identify pipe damage using the Duffing oscillator. Acknowledging the subjective nature of qualitative chaos indices in determining system motion states, some scientists began exploring quantitative chaos indexes. Zhang [25] examined the effectiveness of an enhanced Duffing system for UGW detection in pipelines by altering the nonlinear term of the Duffing equation. Hu [26,27] detected weak second harmonic signals in plates due to micro-cracks by assessing the maximum Lyapunov exponent of the Duffing equation. Wu [28] carried out simulation and experimental studies on the UGW detection of pipeline defects using the maximum Lyapunov exponent and the Lyapunov fractional dimensions as phase determination indexes, respectively. Ng [29] identified hole defects in rails using the maximum Lyapunov exponent of the Duffing equation. Additionally, Cheng [30] developed a pipeline damage detection method based on the double Duffing equation for detecting weak defect echo signals caused by pipeline defects.

Scholars have conducted extensive research on utilizing chaotic oscillators for weak signal detection. However, challenges persist in applying these methods to detect UGW signals:

- The difficulty in the quantitative determination of the system parameters of a chaotic oscillator when it is in the critical state between chaotic and periodic states;
- The commonly employed quantitative measures of chaos, such as maximum Lyapunov exponents and Lyapunov dimensions, necessitate constant re-orthogonalization during calculations, leading to computational inefficiency;
- Currently, only qualitative assessment and localization of damage can be achieved, as it is difficult to quantitatively characterize defects using chaotic oscillators.

Otherwise, piezoelectric acoustic transducers are commonly used in UGW excitation and reception technology for current rail detection. Although the efficiency of the transducer is high, it is strongly influenced by the coupling conditions, which limits its engineering applicability.Thus, it is important to develop a non-contact UGW transducer.

Given the challenges that current studies have struggled to address, this paper aimed to achieve the following research objectives:

1. To develop a quantitative method to determine the system parameters of a chaotic oscillator in the critical state;
2. To develop a computationally efficient quantitative characterization of chaos;
3. To develop a method for quantitative characterization of rail flaws;
4. To design a non-contact UGW transducer.

The outline of this paper is as follows: In Section 2, a UGW signal identification model based on the chaotic oscillator is introduced. The model incorporates a method for calculating Kolmogorov entropy ($K_{e}$) through orthogonal triangular decomposition. Within this framework, $K_{e}$ is employed as a quantitative index that characterizes the motion state of the chaotic oscillator system. In Section 3, an electromagnetic transducer is designed which can achieve unidirectional excitation for UGWs at the rail base and rail head, and experimental verification confirmed that the EMAT successfully amplified forward mode signals and suppressed reverse mode signals. In Section 4, experiments demonstrated that the conventional wavelet transform method is incapable of detecting weak UGW signals reflected by small-size defects. Furthermore, this study’s proposal to use the Kolmogorov entropy of the Duffing oscillator for identifying rail damages was experimentally validated, highlighting its effectiveness in damage identification. Section 5 provides a summary of this study.

## 2. Detection UGW Signal Using a Chaotic Oscillator

### 2.1. Duffing Oscillator System

Since the Duffing equation contains a cosine element and the UGW signal is excited in the form of a trigonometric function, the dynamic system expressed by the Duffing equation is used as a UGW signal detection system.The standard Duffing equation can be expressed mathematically through the state space as follows:

(1) $$\left\{\begin{array}{cc} \frac{d\psi_{1}}{dt} & =\psi_{2}\\ \frac{d\psi_{2}}{dt} & =\psi_{1}-\psi_{1}^{3}-\delta \psi_{2}+\gamma \cos\Omega \psi_{3}+s\left(t\right)\\ \frac{d\psi_{3}}{dt} & =1 \end{array}\right.$$

where δ refers to the damping ratio; γ refers to the amplitude of driving force of the Duffing oscillator; Ω refers to the angular frequency; $\psi_{1}$, $\psi_{2}$, and $\psi_{3}$ correspond to displacement, velocity, and time in state space; and s(t) is the UGW signal to be detected. In numerical calculations, s(t) can be expressed as

(2) $$s\left(t\right)=\frac{A}{2}\left(1-\cos\frac{\omega t}{10}\right)\sin\omega t+\sigma n\left(t\right)$$

In Equation (2), the first term on the right-hand side illustrates the UGW signal modulated by the Hanning window, where A is the amplitude of the guided wave signal, and ω is its angular frequency. The second term represents Gaussian noise, σ refers to the magnitude of the noise, and n(t) is white noise with standard normal distribution.

While Ω=ω, inputting the UGW signal into the Duffing system is equivalent to increasing the amplitude of the driving force. Since the Duffing oscillator is in a critical state between chaotic and periodic motion, increasing the magnitude of the driving force will cause the system to undergo a transition from a chaotic to periodic state.

**Figure 1:**
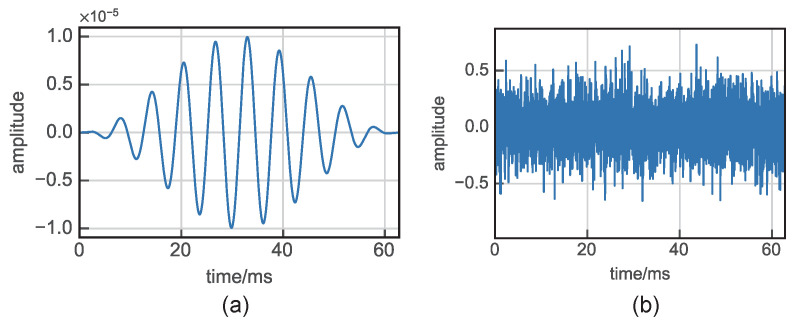
UGW signal and noise signal: (**a**) Guided wave signal where A=0.00001, (**b**) Gauss white noise where σ=0.2.

**Figure 2:**
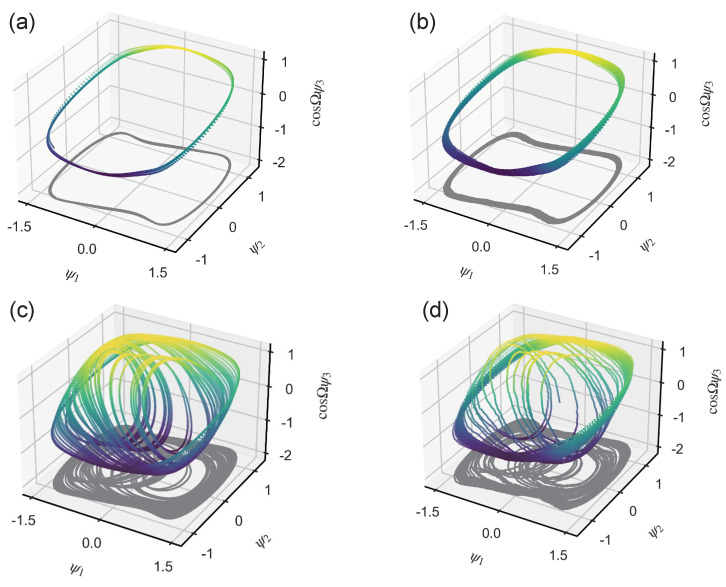
Phase portrait under different input signals: (**a**) when A=0,σ=0, periodic state; (**b**) when A=0,σ=0.2, periodic state; (**c**) when A=0.00001,σ=0, chaotic state; (**d**) when A=0.00001,σ=0.2, chaotic state.

Upon inputting both UGWs and noise signals (as shown in Figure 1) into the Duffing oscillator system, the motion state of the system is scrutinized using the phase portrait, as illustrated in Figure 2. While δ=0.5,γ=0.73493,Ω=ω=1, the Duffing system is in a critical state. Inputting UGW signals of very small magnitude at this point, the motion state of the system will change, while noise is only a perturbation of the system state.

### 2.2. Kolmogorov Entropy as a Quantitative Index of Chaos

Determining the state of motion of the Duffing system from the phase portrait is somewhat subjective, so it is preferable to use a quantitative index to describe its state. Kolmogorov entropy (later referred to as $K_{e}$) is an extended concept of Shannon entropy, which is a measure that describes the degree of chaos in a dynamical system and represents the average information growth of the dynamical system, as shown in Table 1.

The more common calculation of $K_{e}$ is currently approximated by the generalized second-order Renyi entropy via a reconstruction-based vector space solution method, due to the fact that $K_{e}$ is numerically equal to the first-order Renyi entropy [31]. Practical engineering often requires a detection method that does not require a benchmark, and the above solution method is an estimation of $K_{e}$ [32]. In this paper, K entropy is calculated from the perspective that $K_{e}$ is defined as the average growth rate of the amount of information.

Consider the one-dimensional discrete mapping:

(3) $$\varphi_{n+1}=F\left(\varphi_{n}\right)$$

Assuming that the interval of variation of the variable φ is divided into n equal sub-intervals and that φ has equal probability in each sub-interval, if φ is known to be in a certain interval, the amount of information obtained is

(4) $$H\left(\varphi \right)=-\sum_{i=1}^{n}\frac{1}{n}\ln\frac{1}{n}=\ln n$$

The mapping enlarges the interval of variation of the variables by a factor of $F^{\prime }\left(\varphi_{n}\right)$ after each iteration, so that each sub-interval becomes $F^{\prime }\left(\varphi_{n}\right)/n$ after the iteration, and the change in the amount of information in the above mapping system after one iteration is

(5) $$\Delta H\left(\varphi \right)=\sum_{i=1}^{n/F^{\prime }\left(\varphi_{n}\right)}\frac{\left|F^{\prime }\left(\varphi_{n}\right)\right|}{n}\ln\frac{\left|F^{\prime }\left(\varphi_{n}\right)\right|}{n}+\ln n=\ln\left|F^{\prime }\left(\varphi_{n}\right)\right|$$

The average per-iteration information growth over the entire process as the number of iterations tends to infinity is given by

(6) $$K_{e}=\Delta \bar{H}\left(\varphi \right)=\lim_{\lambda \to +\infty }\frac{1}{\lambda }\sum_{i=0}^{\lambda }\left|F_{\left(i\right)}^{\prime }\left(\varphi_{n}\right)\right|$$

For an m-dimensional multidimensional system, the Kolmogorov entropy is

(7) $$K_{e}^{\left(m\right)}=\int \rho \left(\varphi \right)\sum_{i=1}^{m}\Delta {\bar{H}}_{i}d\varphi$$

where ρ(φ) refers to the density of states function in phase space, and for an dynamic system with no concrete physical meaning, the density of states is assumed to be invariant, so that we can obtain

(8) $$K_{e}^{\left(m\right)}=\sum_{i=1}^{m}\Delta {\bar{H}}_{i}\int \rho \left(\varphi \right)d\varphi =\sum_{i=1}^{m}\Delta {\bar{H}}_{i}$$

Thus, the key point in calculating $K_{e}$ is to solve Equation (6). The following focuses on the $K_{e}$ calculation of the Duffing oscillator. The differential equation represented by the Duffing oscillator can be expressed as

(9) $$\dot{\Psi }=J\Psi$$

where J refers to the Jacobi matrix. Performing an orthogonal triangular decomposition of Ψ, i.e., decompose Ψ into a product of an orthogonal matrix and a positive upper triangular matrix; that is,

(10) Ψ=QR

where Q is a orthogonal matrix, R is a positive upper triangular matrix, so we obtain

(11) $$\dot{Q}R+Q\dot{R}=JQR$$

Multiplying the left by the transpose matrix of Q and the right by the inverse matrix of R, we obtain

(12) $$Q^{T}\dot{Q}+\dot{R}R^{-1}=Q^{T}JQ$$

Introducing the intermediate variable θ, the orthogonal matrix Q is denoted as

(13) $$Q=\left(\begin{array}{cc} \cos\theta & \sin\theta \\ -\sin\theta & \cos\theta \end{array}\right)$$

the positive upper triangular matrix R is denoted as

(14) $$R=\left(\begin{array}{cc} e^{\eta_{1}} & r_{12}\\ 0 & e^{\eta_{2}} \end{array}\right)$$

The Jacobi matrix for the Duffing equation is

(15) $$J=\left(\begin{array}{cc} J_{11} & J_{12}\\ J_{21} & J_{22} \end{array}\right)=\left(\begin{array}{cc} 0 & 1\\ 1-3\varphi_{1}^{2} & -\delta \end{array}\right)$$

Combining the above equations, we obtain

(16) $$\left\{\begin{array}{cc} \frac{d\eta_{1}}{dt} & =J_{11}\cos^{2}\theta -J_{21}\sin\theta \cos\theta -J_{12}\sin\theta \cos\theta +J_{22}\sin^{2}\theta \\ \frac{d\eta_{2}}{dt} & =J_{11}\sin^{2}\theta +J_{21}\sin\theta \cos\theta +J_{12}\sin\theta \cos\theta +J_{22}\cos^{2}\theta \\ \frac{d\theta }{dt} & =-J_{11}\sin\theta \cos\theta -J_{21}\cos^{2}\theta +J_{12}\sin^{2}\theta +J_{22}\sin\theta \cos\theta \end{array}\right.$$

The intermediate variables $\xi_{1},\xi_{2}$ are introduced and satisfy the following equation:

(17) $$\left\{\begin{array}{cc} \frac{d\xi_{1}}{dt} & =\frac{d\eta_{1}}{dt}+\frac{d\eta_{2}}{dt}\\ \frac{d\xi_{2}}{dt} & =\frac{d\eta_{1}}{dt}-\frac{d\eta_{2}}{dt} \end{array}\right.$$

so we can obtain

(18) $$\left\{\begin{array}{cc} \frac{d\xi_{1}}{dt} & =J_{11}+J_{22}\\ \frac{d\xi_{2}}{dt} & =\left(J_{11}-J_{22}\right)\cos2\theta -\left(J_{12}+J_{21}\right)\sin2\theta \\ \frac{d\theta }{dt} & =\frac{1}{2}\left(J_{22}-J_{11}\right)\sin2\theta -J_{21}\cos^{2}\theta +J_{12}\sin^{2}\theta \end{array}\right.$$

Substituting the Jacobi matrix of the Duffing system into the above equation, we can obtain

(19) $$\left\{\begin{array}{cc} \frac{d\xi_{1}}{dt} & =-\delta \\ \frac{d\xi_{2}}{dt} & =(3\psi_{1}^{2}-2)\sin2\theta +\delta \cos2\theta \\ \frac{d\theta }{dt} & =-\frac{1}{2}\delta \sin2\theta +\left(3\psi_{1}^{2}-2\right)\cos^{2}\theta +1\\ \frac{d\eta_{1}}{dt} & =\frac{1}{2}\left(\frac{d\xi_{1}}{dt}+\frac{d\xi_{2}}{dt}\right)\\ \frac{d\eta_{2}}{dt} & =\frac{1}{2}\left(\frac{d\xi_{1}}{dt}-\frac{d\xi_{2}}{dt}\right) \end{array}\right.$$

By solving the differential equations Equation (19), the $K_{e}$ can be calculated as:

(20) $$K_{e}=\max\left(\eta_{1},0\right)+\max\left(\eta_{2},0\right)$$

**Figure 3:**
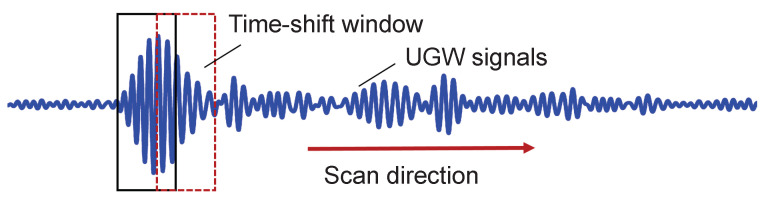
Time-shift window to localize the defect.

To localize the damage, a time-shift window is employed, as illustrated in Figure 3. Only signals falling within this time-shifted window are fed into the critical state Duffing oscillator system after undergoing periodic continuation. Therefore, the presence of a signal within the window with $K_{e}>0$ signifies a damage defect, while the absence indicates no defect.

### 2.3. Determination of the Parameters of the Duffing Equation for the Critical State

To use the Duffing oscillator as a detection system for UGW signals, one of the keys is to set the system as a critical state between chaotic and periodic motions, and it is important to determine the parameters of the damping ratio and driving force amplitude at the critical state. A common method of determining critical state parameters is through bifurcation diagrams. The basic idea is to determine a specific damping ratio, solve the Poincare map of the Duffing oscillators for different amplitudes of driving force, and then the set of projections on the Poincare map is the bifurcation diagram. A bifurcation diagram cannot quantify the degree of chaos and is still essentially a qualitative rather than quantitative assessment method.

It has been shown in Section 2.2 that $K_{e}$ can be used as a quantitative characterization index of the chaotic motion state, and therefore the critical parameter can be determined by the relationship of $K_{e}$ to the change in the motion state of the system with the driving force amplitude γ at different damping ratios δ. In practical engineering terms, it is ideal to be able to find a quantitative indicator that is strictly positively or negatively correlated with the degree of damage.

**Figure 4:**
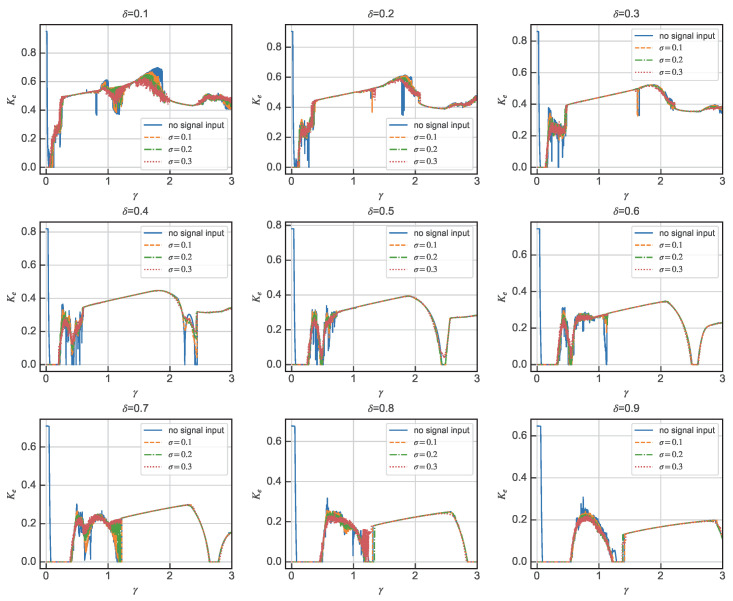
Relating γ and $K_{e}$ for different damping ratios δ when Ω=1.

As is shown in Figure 4, in some damping ratios, there is a large range in which $K_{e}$ is strictly positively correlated with the amplitude of the driving force γ, and the noise has almost no effect on the $K_{e}$ of the system in this range. The UGW signals will be normalized during the actual detection, and the amplitude of the guided wave at the damage is generally smaller than its peak amplitude, so the range of γ from 0.4 to 0.9, and the range of $K_{e}$ strictly increasing with the amplitude of the driving force, generally covers the amplitude of the damaged UGW signals. Considering the diversity and uncertainty of an actual detection environment, we still choose the critical point of the periodic and chaotic states as the driving force amplitude of the detection system, and the critical point is taken as δ=0.8,γ=1.3040. It is worth noting that, in an actual detection, due to the influence of the environment and other signal aberrations and other complex factors, it is impossible to achieve the ideal state of the simulation, and a certain threshold range should be maintained when taking the driving force amplitude. The driving force amplitude is taken in the left field of the critical point, although to some extent it will not be able to achieve an ideal state in the simulation. Although it will reduce the detection sensitivity to some extent, it can improve the stability of the Duffing detection system.

**Figure 5:**
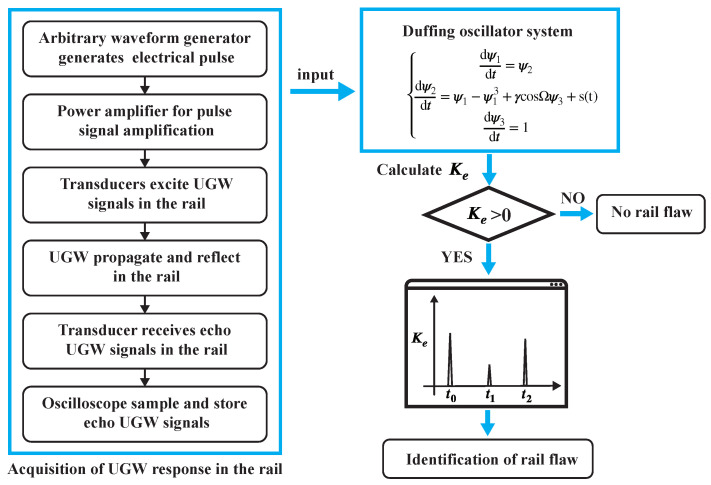
Flow chart of the rail flaw detection based on the Duffing oscillator system.

Thus, a rail flaw detection model based on the Duffing oscillator is established. By evaluating the system’s motion state before and after introducing the UGW response, the identification of ultrasonic guided wave signals can be achieved. $K_{e}$ is a measure that describes the degree of chaos in a dynamical system. A method for calculating $K_{e}$ based on orthogonal triangular decomposition is proposed, making it a quantitative characterization factor of the motion state of the chaotic oscillator system. The entire flow of the above method is shown in the Figure 5.

## 3. Design of an Electromagnetic Acoustic Transducer for UGWs

The ultrasonic acoustic transducers in NDT based on UGWs mainly include piezoelectric, electromagnetic, air-coupled, and laser acoustic transducers. The piezoelectric acoustic transducer is currently the most widely used type, but the disadvantage is that it is greatly affected by the coupling conditions, thus limiting its applicability to engineering sites. Therefore, designing a non-contact type transducer is of great importance for the realization of rail flaw detection.

**Figure 6:**
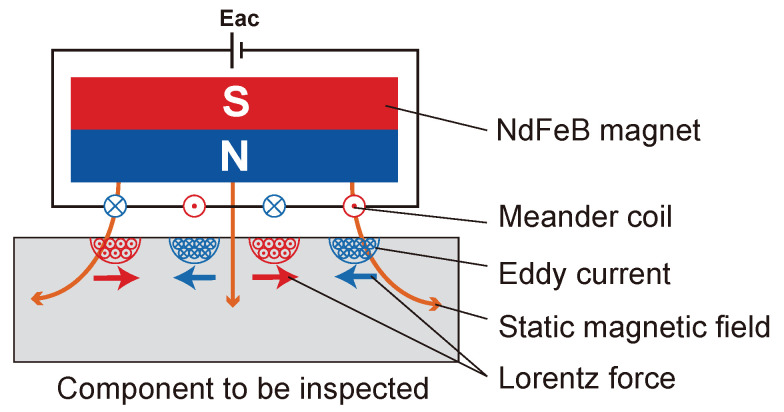
Schematic diagram of UGWs excited by EMAT.

Electromagnetic acoustic transducer(EMAT) has the advantage of good design-ability and lower material costs compared to the lower conversion efficiency of piezoelectric transducers.The principle of the excitation of the Lorentz force-based EMAT is shown in Figure 6.

**Figure 7:**
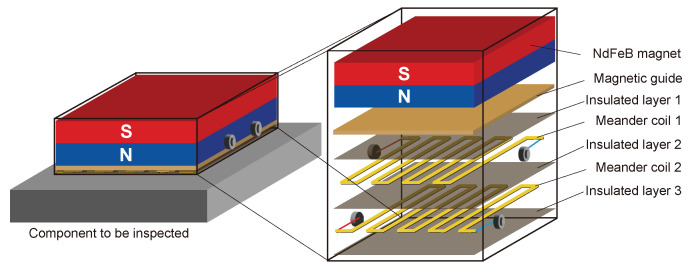
Schematic diagram of EMAT.

**Figure 8:**
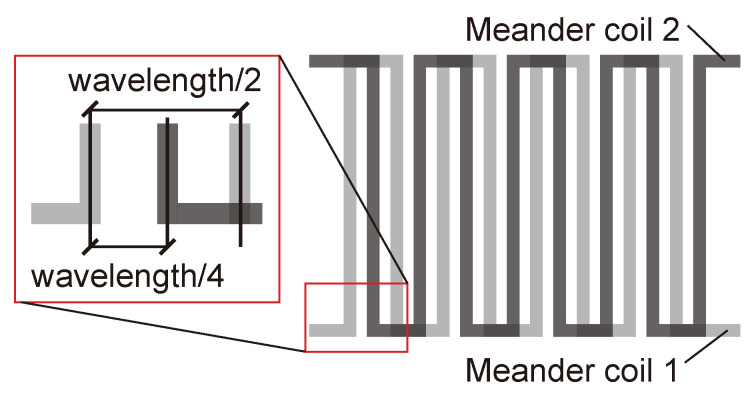
Arrangement of two meander coils.

**Figure 9:**
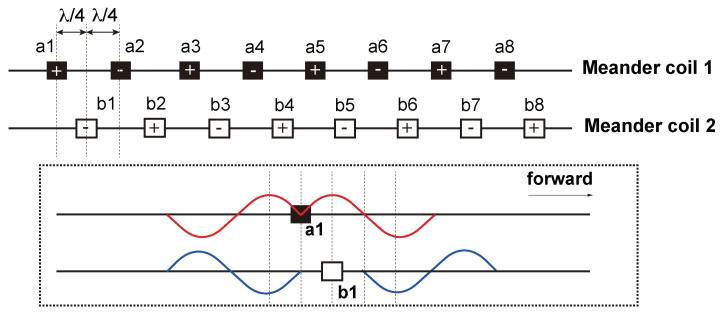
Schematic diagram of the principle of unidirectional guided wave excitation by EMAT.

Due to the complexity of the rail cross-section, it is difficult to excite a single guided wave mode in the rail, and when the guided wave encounters the boundary, the reflection will undergo a complicated mode conversion. If the guided wave propagates from both sides in the rail, the transducer will receive the reflected wave from both sides, which will greatly increase the difficulty of the subsequent signal identification and feature extraction. Therefore, in this study, a unidirectional excitation EMAT is designed to achieve enhancement of the forward guided waves modal and suppression of the reverse modal. The basic components of the EMAT are shown in Figure 7. Amplification in the forward direction and suppression in the backward direction of guided-wave signals is achieved by the arrangement of two meander coils spaced apart from each other and fed with electrical pulses with a time delay, and the arrangement of the two coils is shown in Figure 8. As is shown in Figure 9, the distance between the adjacent wires of the coil is λ/2 (where λ is the wavelength of the guided waves), so that the guided waves generated by each wire gain each other. The distance between coil 1 and coil 2 is λ/4, and a reverse current is applied with a time delay of T/4 (where *T* is the cycle of the guided waves) to achieve amplification of the forward UGW signals and suppression of the reverse signals.

**Figure 10:**
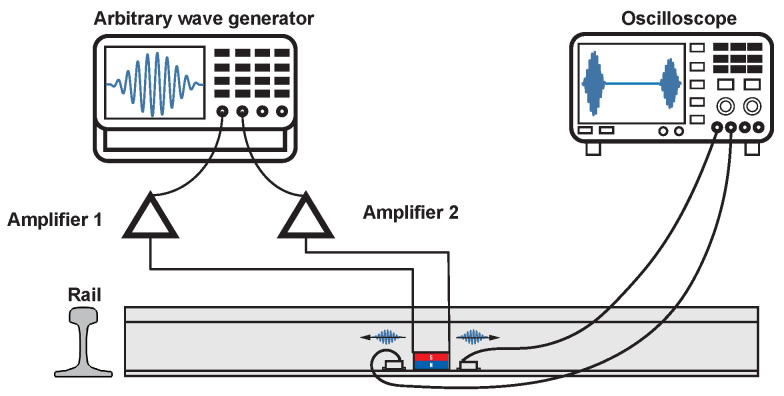
Experiment to verify the effect of the EMAT on the excitation of UGWs in the rail.

**Figure 11:**
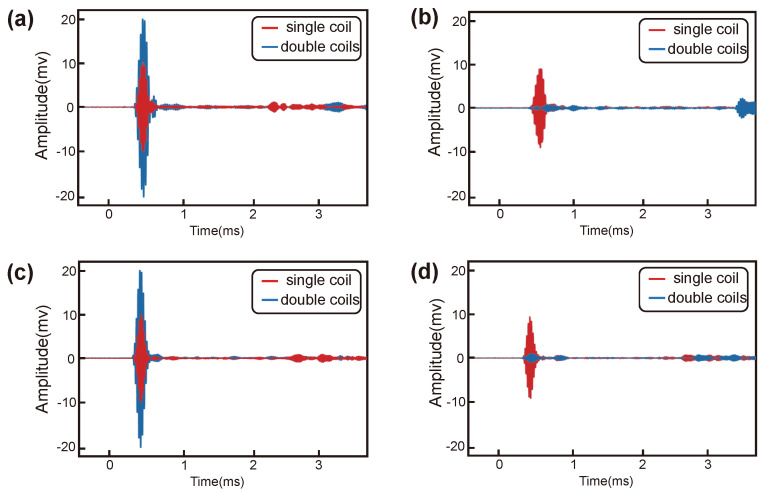
Experimental results: (**a**) Forward UGW signals at the rail base; (**b**) reverse UGW signals at the rail base; (**c**) forward UGW signals at the rail head; (**d**) reverse UGW signals at the rail head.

The effectiveness of the EMAT in exciting UGWs was verified through an experiment at the head and base of the rail. The experimental scheme is shown in Figure 10. The effect of the unidirectional excitation of the double meander coils was verified by comparing the guided wave signals received at equidistant positions on both sides of the EMAT, and the experimental results are shown in Figure 11. The experimental results showed that the EMAT designed in this study could achieve the amplification of the forward guided wave signals and the suppression of the backward guided wave signals at the rail head and at the rail base.

Thus, an EMAT was developed to enable unidirectional excitation of UGW signals at both the rail base and rail head. The strategic placement of two meander coils, separated at a specific distance, allows the amplification of guided-wave signals in the forward direction, while simultaneously suppressing them in the backward direction. This effect is achieved through feeding the coils with electrical pulses that are intentionally time-delayed.

## 4. Experiments to Detect Rail Defects

NDT technology based on UGWs includes four steps: excitation, propagation, reception, and signal processing of UGWs. In order to verify the effectiveness of the NDT method utilizing UGWs based on a chaotic oscillator, an experimental study on rail flaw detection was conducted.

### 4.1. Experimental Design

Based on the above research, experimental schemes for damage detection of rail head and rail bottom were established. First, an arbitrary waveform generator produced a sinusoidal signal modulated by the Hanning window, which was passed through a power amplifier to obtain amplification. The amplified signal was then passed through the EMAT to excite UGWs in the rail. Next, the guided ultrasonic waves were reflected when they encountered defects during propagation in the rail. The reflected guided wave signals were then sampled as electrical signals by an oscilloscope through a receiving acoustic transducer. The sampled guided wave signals were then input into the detection system based on the Duffing oscillator described in Section 2, which ultimately enabled the identification of rail damage.

In the UGW detection experiment, it was important to reduce crosstalk between the external environmental noise and signals in each channel. To achieve this, it is recommended to use BNC radio frequency cables with shielding layers and to keep the cable length as short as possible to minimize signal distortion. Additionally, it is crucial not to share the ground of each unit and to avoid sharing the shield of the signal cable with the ground of other electrical equipment. Furthermore, it is advisable to keep power and signal cables of the equipment as far apart as possible. In cases where separation is not feasible, one should avoid cable crossings, refrain from laying cables in parallel, and if crossing is necessary, do so vertically. Furthermore, it is important to avoid setting the amplifier power too high, as excessive power can result in the generation of odd numbers of high harmonic currents, due to the non-linearity of the electronic circuit.

**Figure 12:**
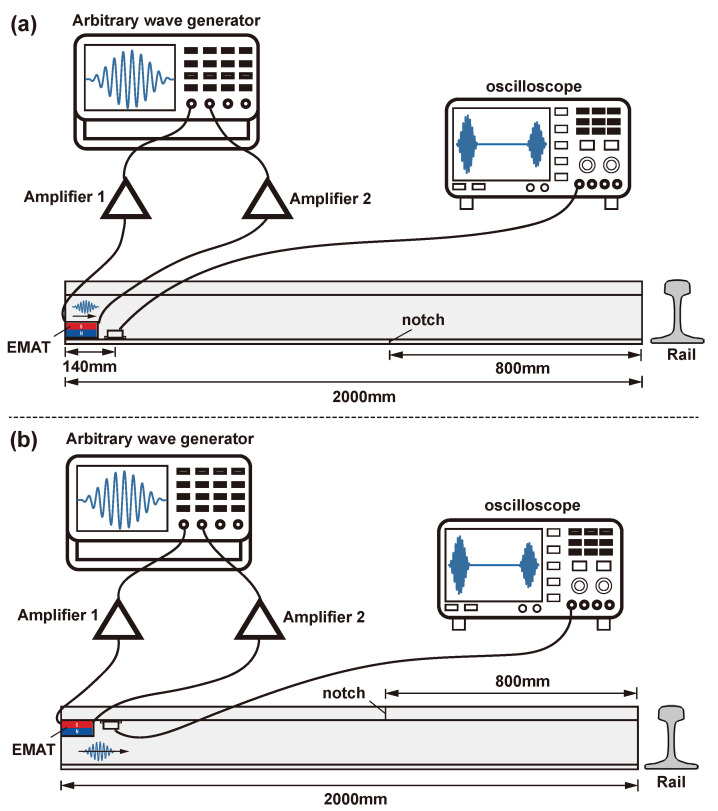
Experimental scheme for rail flaw detecting: (**a**) rail base; (**b**) rail head.

The experimental scheme is shown in Figure 12. For rail base detection, the EMAT was attached to the top of the rail base on both sides. For rail head detection, the EMAT was attached to the bottom of the rail head on both sides.

**Figure 13:**
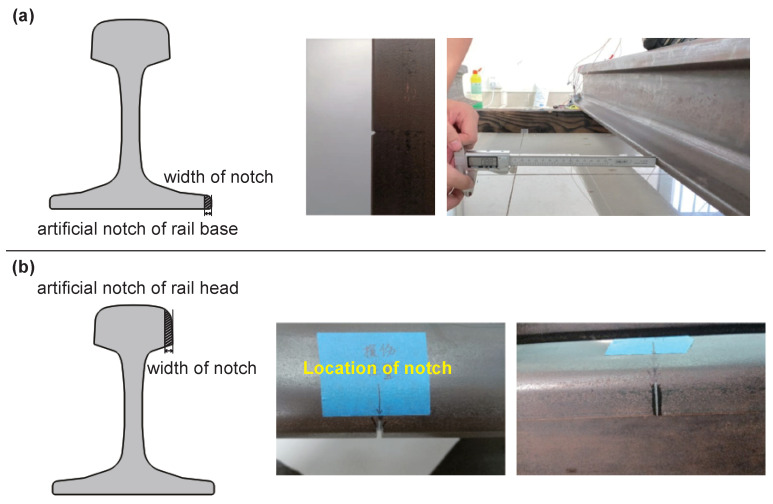
Artificial notches in the experiment: (**a**) rail base; (**b**) rail head.

In the experiment, artificial notches were set at the rail base and rail head, as described in Figure 13. There were 7 different notches for the rail foot and 9 different notches for the rail head. The specific notch sizes for different cases at the rail base can be seen in Table 2, and the specific notch sizes at the rail head can be seen in Table 3.

### 4.2. Experimental Results

**Figure 14:**
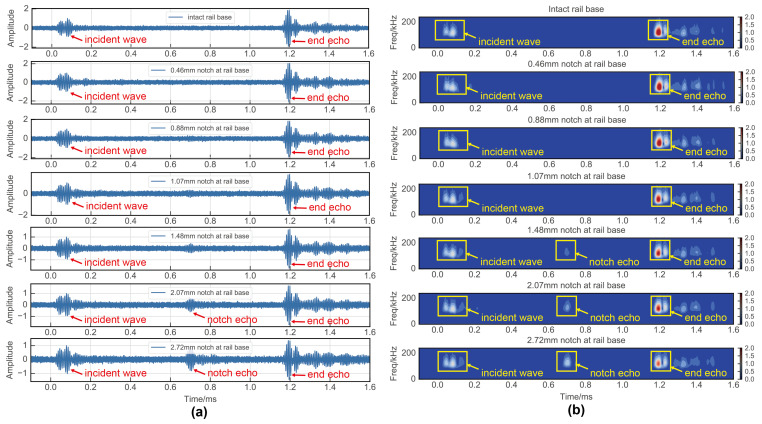
Experimental results of rail base detection: (**a**) time domain signal; (**b**) wavelet transform.

**Figure 15:**
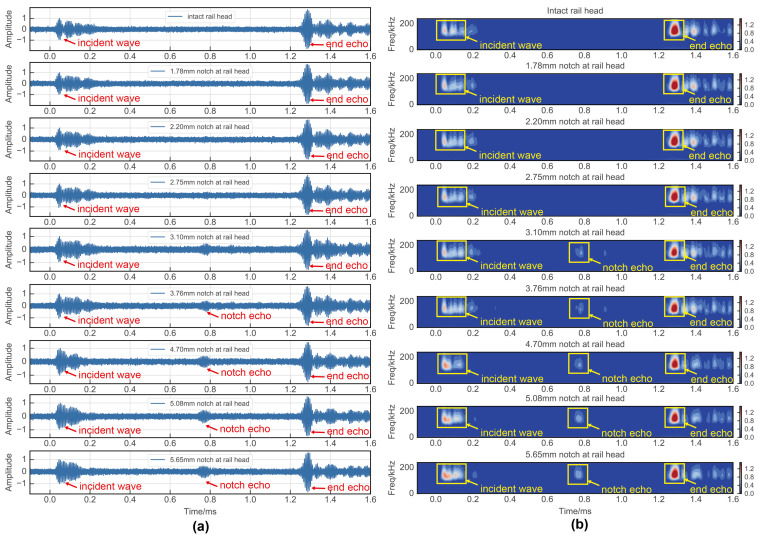
Experimental results of rail head detection: (**a**) time domain signal; (**b**) wavelet transform.

In the flaw detection experiments conducted at the rail base and rail head, the time domain signals of the guided waves were as depicted in Figure 14a and Figure 15a. Initially, it was apparent that the time domain signal alone had limited efficacy in detecting damage, with only a 2.07 mm notch discernible at the rail base and a 3.76 mm notch at the rail head due to smaller defects being overshadowed by noise. Given this challenge, traditional signal processing approaches resort to signal cancellation techniques. Among these techniques, time–frequency analysis stands out as a commonly utilized method in current research practices. To address this limitation, the present study employed time–frequency analyses using a wavelet transform on the UGW signals. The outcomes of this analysis are displayed in Figure 14b and Figure 15b. The application of the wavelet transform yielded a notable noise reduction effect, thereby enhancing the damage detection threshold to some extent. Nevertheless, the method still faced challenges in detecting minute damages. Specifically, the wavelet transform-based time–frequency analysis method successfully identified a 1.48 mm notch at the rail base and a 3.10 mm notch at the rail head.

**Figure 16:**
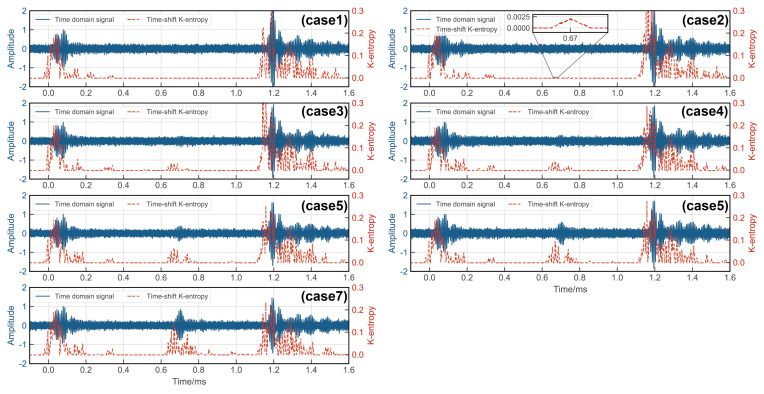
Experimental results of rail base detection via $K_{e}$ of Duffing oscillator.

**Figure 17:**
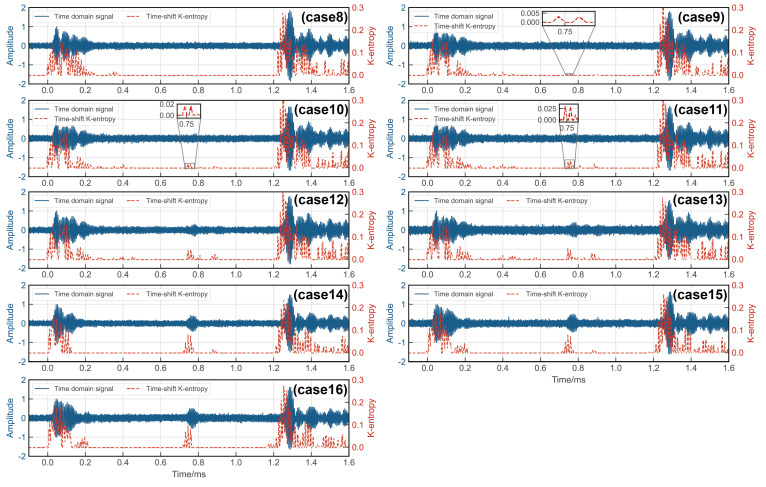
Experimental results of rail head detection via $K_{e}$ of Duffing oscillator.

Rail damage was detected using the chaotic oscillator detection system as discussed in Section 2. The incoming UGW signals were processed by the critical Duffing oscillator system, with the parameter $K_{e}$ serving as an indicator of the system’s motion state. To localize the damage, a time-shift window was employed. Only signals falling within this time-shifted window were fed into the critical state Duffing oscillator system after undergoing periodic continuation. Therefore, the presence of a signal within the window with $K_{e}>0$ signified a damage defect, while the absence indicated no defect. The results are depicted in Figure 16 and Figure 17, revealing the system’s ability to detect a 0.46 mm notch at the rail base and a 1.78 mm notch at the rail head. Upon comparing the wavelet transform method with the proposed method, it is evident that the method introduced in this paper demonstrated greater sensitivity to rail damage and enhanced the threshold for detecting rail flaws.

The results of identifying and localizing rail damage using the $K_{e}$ of the Duffing oscillator system are presented in Table 4 and Table 5.

The position error ($p_{e}$) for the rail notch could be calculated using Equation (21) as

(21) $$p_{e}=\left|\frac{\left(L-l_{0}\right)\left(t_{2}-t_{1}\right)}{l_{1}\left(t_{1}-t_{0}\right)}-1\right|$$

where *L* is the axial length of the rail, $l_{0}$ is the axial distance between the center of the receiver transducer and the excited end face of the rail, $l_{1}$ is the actual axial distance between the center of the receiver transducer and the rail notch, $t_{0}$ represents the time of the incident wave with maximum amplitude, $t_{1}$ corresponds to the time of the end echo with maximum amplitude, and $t_{2}$ is the time corresponding to the maximum amplitude of notch echo.

**Figure 18:**
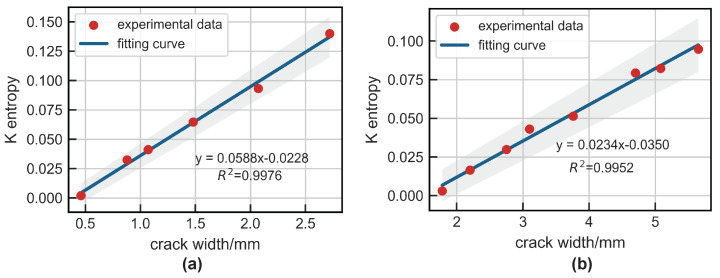
The relationship between notch width and $K_{e}$: (**a**) rail base; (**b**) rail head.

Moreover, the width of notches at the rail base and rail head were directly proportional to the $K_{e}$, as shown in Figure 18. Thus, Kolmogorov entropy could be used as a quantitative index to characterize the rail defects. The experimental results demonstrated the effectiveness of the detection system based on a Duffing oscillator for rail flaw detection. Specifically, the Duffing oscillator system was capable of detecting a 0.46 mm notch at the rail base and a 1.78 mm notch at the rail head.

## 5. Conclusions and Discussion

This study addressed the challenge of identifying weak UGW signals in a strong noise background in rail flaw detection by proposing a damage identification method based on chaotic oscillators. Initially, a mathematical model for detecting UGW signals using the Duffing oscillator was introduced. The motion state of the Duffing system was characterized by the Kolmogorov entropy, and a formula for calculating this entropy was established. Subsequently, an electromagnetic UGW transducer was developed to amplify forward UGW modes and suppress unidirectional UGW modes. The efficacy of the proposed model and transducer in rail damage detection was then validated through experimental testing. The main conclusions of this study are analyzed as follows:

1. A UGW signal identification model based on the chaotic oscillator was established. The approach integrates the UGW response into the critical state of the Duffing system to serve as a disturbance control variable. This incorporation leads to alterations in the system’s motion state through the exploitation of the parameter disturbance sensitivity characteristic of chaotic systems and the traversal of chaotic motion. By evaluating the system’s motion state both pre- and post-introduction of the UGW response, the identification of ultrasonic guided wave signals can be realized. This methodology encapsulates the fundamental concept of employing chaotic systems for discerning faint guided wave signals in NDT applications centered on UGWs;
2. A method for calculating Kolmogorov entropy based on orthogonal triangular decomposition was proposed. Kolmogorov entropy is a measure that describes the degree of chaos in a dynamical system and represents the average information growth of the system. $K_{e}$ can be used as a quantitative characterization factor of the motion state of the chaotic oscillator system. When $K_{e}=0$, the system is in a state of periodic motion, and when $0<K_{e}<+\infty$, the system is in a chaotic state. This method eliminates the need for reconstructing the phase space, thereby improving the efficiency of calculating Kolmogorov entropy.
3. An electromagnetic transducer was designed that can achieve unidirectional excitation for UGWs at the rail base and rail head. Amplification in the forward direction and suppression in the backward direction of guided-wave signals was achieved though the arrangement of two meander coils spaced apart from each other and fed with electrical pulses with a time delay.The distance between adjacent wires of the coil was λ/2, so that the UGW generated by each wire gained each other. The distance between coil 1 and coil 2 was λ/4, and a reverse current was applied with a time delay of T/4 to achieve amplification of the forward guided wave signal and suppression of the reverse signal. Experimental verification confirmed the effectiveness of the EMAT in producing the desired effects mentioned above;
4. The experimental results indicated the challenge in effectively identifying the weak UGW echoes caused by small sized damage using time-domain signals. Although the traditional signal processing method based on wavelet transform showed improved denoising capabilities, it continued to struggle in effectively distinguishing the weak UGW signals.
5. The width of notches at both the rail base and rail head were directly proportional to the $K_{e}$, hence Kolmogorov entropy can serve as a quantitative characterization index of rail damage. The experimental results demonstrated the effectiveness of the detection system based on a chaotic oscillator in detecting weak UGW signals. Specifically, the Duffing oscillator system was capable of detecting a 0.46 mm notch at the rail base and a 1.78 mm notch at the rail head.

In summary, this study proposed a method for detecting rail flaws using the Kolmogorov entropy of a chaotic oscillator based on UGWs. This method aims to accurately locate and quantitatively characterize defects at the rail base and rail head to enhance the sensitivity of rail flaw detection. However, in engineering applications, the method described above may encounter limitations, particularly when dealing with large rail damage. In such cases, the guided wave within the damaged area may undergo mode conversion. The presence of multiple damages on the rail further complicates the situation, making it challenging to differentiate between the modal conversion signal of the initial damage and the reflection signal produced by subsequent damages. Hence, the study of the specific interaction between rail flaws and UGWs remains a key research direction for the future application of the method proposed in this paper.

## Figures and Tables

**Table 1:** A criterion for determining the motion state of a dynamical system by means of $K_{e}$.

The Value of K	Motion State
$K_{e}=0$	periodic state
$0<K_{e}<+\infty$	chaotic state
$K_{e}=+\infty$	complete random state

**Table 2:** Various cases of defects at the rail base.

Case	Case 1	Case 2	Case 3	Case 4	Case 5	Case 6	Case 7
Notch width/mm	0	0.46	0.88	1.07	1.48	2.07	2.72

**Table 3:** Various cases of defects at the rail head.

Case	Case 8	Case 9	Case 10	Case 11	Case 12	Case 13	Case 14	Case 15	Case 16
Notch width/mm	0	1.78	2.20	2.75	3.10	3.76	4.70	5.08	5.65

**Table 4:** Results of locating the notch at the rail base.

Case Number	Width of Notch/mm	$t_{0}$ (ms)	$t_{1}$ (ms)	$t_{2}$ (ms)	$K_{e}$	Positioning Error (%)
1	0	0.038732	1.158122	/	0	/
2	0.46	0.038732	1.158122	0.670047	0.001992	1.04
3	0.88	0.038732	1.142207	0.670047	0.032303	0.39
4	1.07	0.038732	1.158122	0.670047	0.041133	1.04
5	1.48	0.038732	1.158122	0.670047	0.064721	1.04
6	2.07	0.038732	1.158122	0.670047	0.093227	1.04
7	2.72	0.038732	1.158122	0.670047	0.139945	1.04

**Table 5:** Results of locating the notch at the rail head.

Case Number	Width of Notch/mm	$t_{0}$ (ms)	$t_{1}$ (ms)	$t_{2}$ (ms)	$K_{e}$	Positioning Error (%)
8	0	0.027364	1.250584	/	0	/
9	1.78	0.027364	1.250584	0.745835	0.003014	3.07
10	2.20	0.027364	1.250584	0.745835	0.016560	3.07
11	2.75	0.027364	1.250584	0.745835	0.029862	3.07
12	3.10	0.027364	1.250584	0.754930	0.042997	4.37
13	3.76	0.027364	1.250584	0.745835	0.051369	3.07
14	4.70	0.031911	1.255131	0.745835	0.079399	2.41
15	5.08	0.031911	1.255131	0.670047	0.082339	2.41
16	5.65	0.031911	1.255131	0.670047	0.094784	2.41

## Data Availability

The data generated during this study are currently private due to pending patent applications.

## Abbreviations

The following abbreviations are used in this manuscript:

UGW	Ultrasonic guided wave
NDT	Non-destructive testing
EMAT	Electromagnetic acoustic transducer
